# New methods for the diagnosis and monitoring of cognitive function in patients with type 2 diabetes

**DOI:** 10.3389/fendo.2022.1024794

**Published:** 2022-12-01

**Authors:** Andreea Ciudin, Rafael Simó

**Affiliations:** ^1^ Endocrinology and Nutrition Department, Hospital Universitari Vall d’Hebron, Vall d’Hebron Institut de Recerca (VHIR), Barcelona, Spain; ^2^ Universitat Autónoma de Barcelona, Department of Human Physiology and Inmunology, Barcelona, Spain; ^3^ CIBER de Diabetes y Enfermedades Metabólicas Asociadas (CIBERDem), Instituto de Salud Carlos III, Madrid, Spain; ^4^ Universitat Autónoma de Barcelona, Department of Medicine, Barcelona, Spain

**Keywords:** type 2 diabetes, mild cognitive impairment, dementia, retinal microperimetry, neuropsychological tests

## Abstract

The presence of type 2 diabetes acts as an accelerator of cognitive impairment (mild cognitive impairment and later dementia), with a significant impact on the management of the disease and its complications. Therefore, it is recommended to perform an annual evaluation of cognitive function in patients with diabetes older than 65 years. Current guidelines still recommend the use of the Minimental State Evaluation Test (MMSE) as screening test, but it has a modest sensitivity and specificity for identifying mild cognitive impairment. This represents an important gap because patients with mild cognitive impairment are at risk of progressing to dementia. The neurocognitive diagnosis is based on complex neuropsychological tests, which require specifically trained personnel and are time consuming, making its routine incorporation into daily clinical practice unfeasible. Therefore, at present there are no reliable biomarkers to identify patients with type 2 diabetes at increased risk of developing cognitive impairment. Since the brain and the retina have a common embryological origin, our Research Group, has worked over the last 10 years evaluating the usefulness of the retina as a “window” to the brain. We provided evidence that retinal microperimetry is a simple, feasible and useful tool for screening and monitoring cognitive function in patients with type 2 diabetes. We propose a review of actual tests recommended for screening of cognitive impairment as well as an update of new emerging methods, such as retinal microperimetry.

## 1 Introduction. State of the art

### 1.1 Type 2 diabetes as risk factor and accelerator for cognitive impairment

Current epidemiological data show that type 2 diabetes (T2D) increases 2-3 times the risk of developing dementia, in comparison with non-diabetic population matched by age and other risk factors (1).

**Figure 1:**
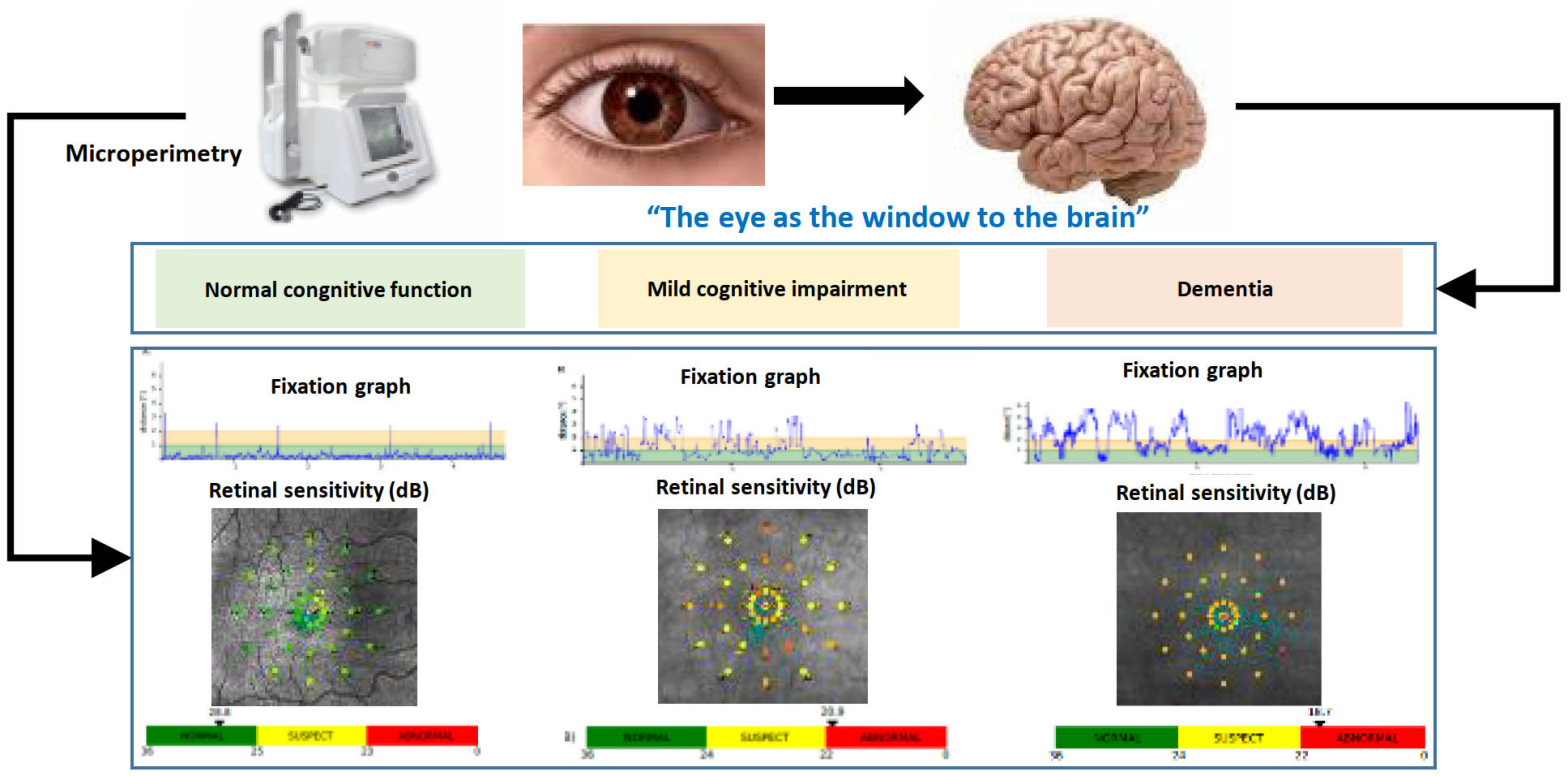
Common mechanisms shared by Type 2 diabetes and Cognitive impairment.

T2D and dementia, in particular Alzheimer´s disease (AD), share several common pathophysiological mechanisms. Both, in T2D and Alzheimer´s disease insulin resistance, low-grade inflammation, oxidative stress, high levels of angiotensin-II and advanced end-glycation products (AGE) are found and contribute to the progression of the disease. Furthermore, a common genetic background was described- Additionally, patients with T2D have smaller brain volumes and white matter disruption as a result of this pathophysiological mechanisms- as reflected by **Figure 1** (2–5).

On these bases we hypothesized that T2D could act as an accelerator of cognitive decline in two ways: by inducing a slow cognitive decline, also called “dementia related to Diabetes”, or by precipitating a rapid cognitive decline to AD, in the presence of predisposing genetic factors, like APOEϵ4 allele (3). Additionally, the presence of complications related to diabetes (micro and macrovascular), as well as metabolic control and the episodes of hypoglycemia will accelerate the progression of cognitive impairment (6–10).

There is a gradual progression from the so-called subjective memory complaints (SMC) to mild cognitive impairment (MCI), which is the prelude to dementia. MCI represents a deterioration in cognition superior to that observed with normal cognitive decline associated with age, but not severe enough to cause significantly impaired daily function (11)

Data in the literature showed that about 33% of women and 20% of men over 65 years of age with MCI could develop dementia throughout during their lifespan (12). Recent data showed that T2D is an independent risk factor, besides the presence of the APOEε4 allele for conversion to dementia in patients with MCI (1). The presence of MCI can lead to mismanagement of the specific treatment for T2D. This can favor episodes of severe hypoglycemia, thus worsening the patients’ cognitive status, all of which translates into an increase in direct and indirect healthcare costs (13, 14). Additionally, hypoglycemia can alter the cognitive function and accelerates the progression to dementia (15–17). For this reason, it is important to diagnose cognitive impairment in early stages in patients with T2D.

### 1.2 Actual recommendation for screening for cognitive impairment in T2D patients

Since 2018, the ADA clinical guidelines started to recommend screening for early detection of cognitive impairment for adults≥ 65 years of age with T2D at the initial visit and annually as appropriate (18, 19). The current guideline (19), recommend for this purpose screening tools such as the Mini Mental State Examination (MMSE) and the Montreal Cognitive Assessment (MoCA), especially in those patients in whom dementia is suspected. Additionally, annual monitoring is indicated for adults >65years with T2D for early detection of mild cognitive impairment or dementia.

#### 1.2.1 The mini mental state examination test

The MMSE, 10-15 minutes test (20)is a test largely used to screen for cognitive impairment. A standard MMSE cut score of 24 (≤23) yielded a sensitivity of 0.58 and a specificity of 0.98 in detecting probable and possible Alzheimer’s disease. A cut score of 27 of a maximum 30 (≤26) resulted in an improved balance of sensitivity and specificity (0.79 and 0.90, respectively). In MCI group the standard cut-off score of 24 yielded a sensitivity of 0.38 and a specificity of 1.00 while raising the cut score to 27 resulted in an improved balance of 0.59 and 0.96 of sensitivity and specificity, respectively (20) (21). Nevertheless, it should be noted that these data are supported on limited number of patients. A recent meta-analysis (22), evaluated the usefulness of MMSE for the detection of dementia in people with MCI. The conclusion, based on 11 studies that meet the accuracy criteria, was that there no reliable evidence to support a substantial role of MMSE in identifying those patients with MCI at risk of developing dementia. The data from the clinical studies that were evaluated is heterogeneous regarding the optimal cut-off, the influence of educational background or the effect of literacy in the accuracy of MMSE.

Regarding the annual monitoring, the same meta-analysis showed a specificity of 88% and sensitivity of 40% in detecting progression of MCI (22).

#### 1.2.2. The Montreal cognitive assessment test

The MoCA test (23), a 10-15 minutes is the other neurocognitive test recommended for screening of cognitive function in patients with T2D>65years. The cut-off of >26 out of maximum 30 identified patients with MCI with a sensitivity of 91% and specificity of 90%. This test needs previous training and periodical certification of the personnel. Furthermore, the result is influenced by the educational level and the mood of the subject.

A recent study (24) showed that MoCA is superior to MMSE in detecting patients at higher risk of dementia. Nonetheless, it should be noted that this conclusion is based on small sample size (<100 subjects in total) and different cut-offs were used (19-20 for MoCA and 23-24 for MMSE).

Additionally, studies that evaluated the usefulness of both MMSE or MoCA excluded patients with depressive symptoms, evaluated by specific questionnaires, such as geriatric depression scale (GDS) or medical history (22, 24). This represents an important limitation because the associated anxiety-depression and impaired quality or life (QoL) occur in a significant proportion of ageing T2D patients. This is important because, apart from influencing the result of neuropsychological tests, the psychological abnormalities related to a decrease in QoL in T2D patients could play a role in aggravating their cognitive decline. In fact, depression was associated with cognitive decline in the ACCORD-MIND study (25). Additionally, the prevalence of depression is two-fold higher in T2D compared with the general population worldwide (26) and it has recently been reported of 27,5% among T2D patients in the Mediterranean population (27) This prevalence rate could even be higher in the older adults with diabetes because depressive symptoms may be overlooked (27).

On these bases, we should raise serious questions about the accuracy and the applicability of these recommended screening tests at large scale in the population with T2D >65 years, especially if we intent to detect cognitive impairment in early stages.

#### 1.2.3. Limitations of the actual recommended tests for screening

- Low sensitivity and specificity for detecting early stages of cognitive impairment
- Results based on studies with small sample size, heterogeneous cut-offs, patients with depressive symptoms were excluded.
- Depend on the educational level and the mood of the patient.

The current guidelines recommend screening of cognitive impairment *especially in those patients in whom dementia is suspected* (28). Nonetheless, the early recognition of cognitive impairment is crucial for a personalized medicine and is an essential issue in diabetes care. The early detection of mild cognitive impairment or even subjects with SMC at risk of progression is important because T2D patients with cognitive impairment are more prone to present an impaired self-management of diabetes, poor glycemic control and an increased incidence of diabetes-related complications. In this regard, the actually recommended tools have serious limitations and at present there is gap due to the lack of rapid, reliable tools for early detection of cognitive impairment in patients with T2D >65years, regardless of the mood or educational level.

## 2 New emerging methods for screening and monitoring of cognitive function

In recent years new methods have emerged for the screening and monitoring of the congnitive function in patients with T2D>65years: the Diabetes Specific Dementia Risk Score (DSDRS) (29) and retinal microperimetry (30).

### 2.1 Diabetes specific dementia risk score

DSRDS was proposed in 2013 by Exalto et al. (29) as a risk score for the prediction of 10-year dementia risk in patients with T2D. This score consists of several clinical and demographic variables (age, gender, education, history of diabetic foot, acute metabolic events, depression, microvascular disease, cardiovascular disease and cerebrovascular disease) and ranges from a 5% 10-year dementia risk for those with the lowest score up to a 73% risk for the highest score.

The DSDRS was not initially designed as a screening tool. Recently, as part of a European project (31),MOPEAD study (32), our research group evaluated the usefulness of the DSDRS as a screening tool for MCI, alone or combined with MMSE. The cognitive status of the patients was confirmed by neuropsycholgical tests battery performed by trained neuropsychologists at the memory clinic. The results showed that a cut-off of DSDRS≥7 had a predictive value of AUC 0.739 (CI95%, [0.557-0.921]) and of 0.902 (CI 95% [0.840-0.992]) when combined with MMSE (33), suggesting that DSRDS could be a reliable screening tool for cognitive decline in patients with T2D>65 years with a sensitivity 88.37% CI95% (78.9-97.9) and specificity 55.26% CI 95% (48.9-60.5). Additionally, a recent study from another group studied the utility of the DSDRS for lower cognitive performance and found similar results, with a sensitivity of 83.3% and specificity of 54.3% (31).

### 2.2 Retinal microperimetry

The retina is ontogenically a brain-derived tissue and it has been suggested that it may provide an easily accessible and non-invasive way of examining the pathology of the brain: “the eye as a window of the brain”. Retinal microperimetry, is a simple, rapid and non-invasive test that measures retinal sensitivity in terms of the minimum light intensity that patients can perceive when spots of light stimulate specific areas of the retina, and also evaluate gaze fixation stability (30, 34).

**Figure 2:**
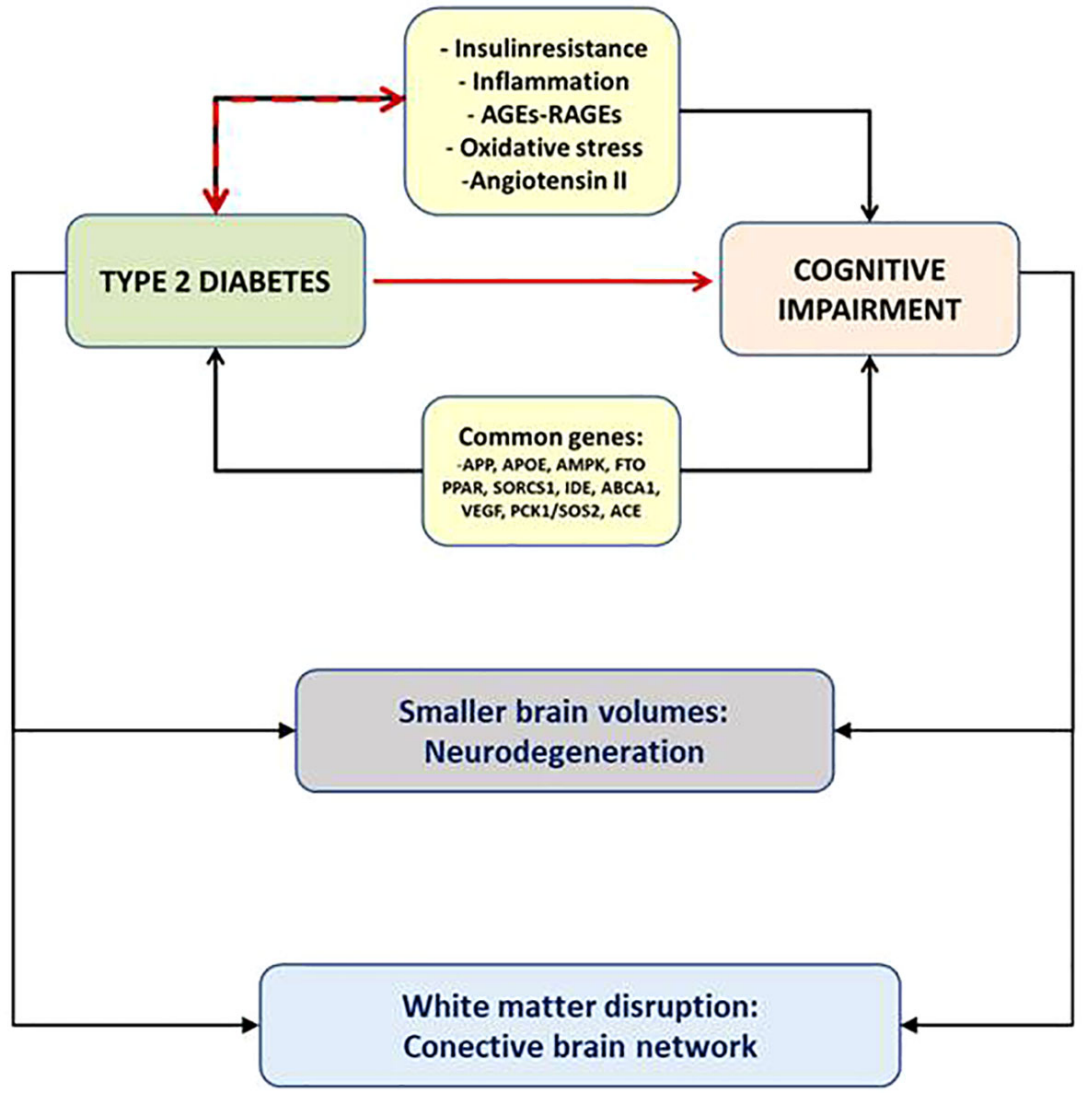
The eye as the window to the brain.

We showed for the first time that the retinal sensitivity significantly correlated with brain imaging (MRI and PET) and identified patients with MCI and dementia, as confirmed by complete neuropsychological battery- **Figure 2**.

By adding the gaze fixation parameters to the retinal sensitivity the probability to identify cognitive impairment significantly increased in an independent manner, suggesting that retinal sensitivity and gaze fixation explore different neuronal circuits and are complementary (35), converting the retinal microperimetry into a potential useful tool for the screening of MCI in patients with T2D >65 years (with a sensitivity of 72.7% and sensibility of 87.9%).

Furthermore, we have recently evaluated the usefulness of retinal microperimetry as a monitoring tool for the cognitive function in patients >65 years with T2D (36). We found that the worsening in cognitive function went in parallel with worsening of gaze fixation, suggesting this parameter can be a reliable and more subtle tool for the monitoring of the cognitive function in patients with type 2 diabetes. These results suggest that since retinal sensitivity is a reliable screening tool for diagnosis, the evaluation of gaze fixation could represent a better biomarker for annual follow-up. As previously explained (35), this finding could be attributed to the fact that the brain areas involved in gaze fixation are not the same as in retinal sensitivity. Retinal sensibility relies on the retina and the neural pathway of vision, which includes the geniculate body and correlated with grey matter volumes in brain MRI in T2D patients with MCI and AD but not white matter (30), suggesting that could be a marker of neurodegeneration. On the other hand, gaze fixation depends on the complex white matter network (37).

It should be noted that fixation eye movements are affected by attention and working memory (38). In this regard, micro-saccade rates transiently decrease during an attentional task (39).The omnipause neurons (located in the nucleus raphe interpositus of the paramedian pontine reticular formation) or the superior colliculus (SC) are two of the main brain areas involved in fixation. It is possible that these areas are affected early in the neurodegenerative process (40). While performing the retinal microperimetry, the subjects are asked to maintain their gaze fixated on a central target, while stimuli with different intensities are presented at 37 points in 3 concentric circles of 2, 6, and 10 degrees of diameter (41). It could be hypothesized that subjects with cognitive impairment are unable to inhibit the saccades triggered by the most eccentric stimuli and maintain their gaze fixated on a central target. Furthermore, certain areas of the cerebral cortex, such as the parietal and frontal cortex (frontal eye fields and the dorsomedial prefrontal cortex), show elevated firing rates during fixation. These areas could contribute to the control of fixation through descending projections to the SC and the omnipause region (40).

Retinal microperimetry, used in the daily clinical practice, as part of the ophthalmological evaluation, is a simple, objective and rapid test that can be largely used for the monitoring of the cognitive performance, regardless of the educational level. Nevertheless, at present retinal microperimetry as a tool for the screening and monitoring of cognitive function has some limitations. First, the presence of advanced DR might be a limiting factor. However, we propose it as tool for early detection of cognitive impairment and at this stage a large percentage of patients with T2D present only mild or moderate DR. Secondly, the device might not be widely available in many primary or even tertiary care settings. Third, although it seems that the test is not influenced by psychological or mood status, at present there are no studies specifically aimed at evaluating this issue. For all these reasons we have to still consider retinal microperimetry as an emerging and promising tool that needs further validation and cost-effectiveness analysis.

These preliminary data, both on DSDRS and microperimetry have the potential of changing the current guidelines of clinical practice in patients with T2D if the results are confirmed in larger clinical trials. In this regard, a European project is ongoing (RECOGNISED study, NCT04281186), aimed at validating the usefulness of retinal microperimetry and DSRDS as reliable screening and monitoring tools for cognitive impairment in patients with T2D >65 years. These two tests can be easily implemented and applied at large scale in the daily clinic. If our results are confirmed, the DSDRS could be a screening tool that might easily be implemented and automatically calculated by the electronic medical records of the patients, as part of the daily clinical practice. Additionally, for the annual microperimetry performance in the ophthalmology clinic, an automatic software could provide reliable data on the patients with T2D>65 years that present significant changes in gaze fixation and retinal sensitivity annually, as possible patients at higher risk of progression of their cognitive impairment, on which diabetes care providers should focus and make an accurate diagnosis and management.

## 3 Concluding remarks

Cognitive impairment is an emerging condition in T2D but often remains undiagnosed due to lack of simple tools for screening at large scale. This is important because T2D patients with cognitive impairment are more prone to present an impaired self-management of diabetes, poor glycemic control and an increased incidence of diabetes-related complications. Unfortunately, current guidelines still recommend the use of tests that depend on the mood of the patient and the educational level, with modest sensitivity and specificity for identifying mild cognitive impairment. This represents an important gap because patients with mild cognitive impairment are at risk of progressing to dementia and the early and reliable identification at large scale will allow the implementation of a patient-centered treatment based on simplifying the regimes and prioritizing antidiabetic treatments with low capacity to provoke hypoglycemia. Recently, new methods have emerged as reliable tools for the cognitive evaluation in patients with T2D>65 years, such as DSRDS and more importantly, retinal microperimetry, that have the potential to change the current guidelines if preliminary results are confirmed in a large ongoing European trial (RECOGNISED).

## Author contributions

AC: Conceptualization, first draft writing, revision. RS: Supervision, draft revision. All authors contributed to the article and approved the submitted version.

## Conflict of interest

The authors declare that the research was conducted in the absence of any commercial or financial relationships that could be construed as a potential conflict of interest.

## Publisher’s note

All claims expressed in this article are solely those of the authors and do not necessarily represent those of their affiliated organizations, or those of the publisher, the editors and the reviewers. Any product that may be evaluated in this article, or claim that may be made by its manufacturer, is not guaranteed or endorsed by the publisher.
